# Comparative Signatures of Selection Analyses Identify Loci Under Positive Selection in the Murrah Buffalo of India

**DOI:** 10.3389/fgene.2021.673697

**Published:** 2021-10-19

**Authors:** Shiv K. Tyagi, Arnav Mehrotra, Akansha Singh, Amit Kumar, Triveni Dutt, Bishnu P. Mishra, Ashwni K. Pandey

**Affiliations:** ^1^ Animal Genetics Division, ICAR-Indian Veterinary Research Institute, Izatnangar, Bareilly, India; ^2^ Livestock Production and Management, Indian Council of Agricultural Research (ICAR)-Indian Veterinary Research Institute, Bareilly, India; ^3^ Animal Biotechnology, Indian Council of Agricultural Research (ICAR)-Indian Veterinary Research Institute, Bareilly, India

**Keywords:** ddRAD, genotypes, bubalus, Fst, XP-EHH

## Abstract

India is home to a large and diverse buffalo population. The Murrah breed of North India is known for its milk production, and it has been used in breeding programs in several countries. Selection signature analysis yield valuable information about how the natural and artificial selective pressures have shaped the genomic landscape of modern-day livestock species. Genotype information was generated on six buffalo breeds of India, namely, Murrah, Bhadawari, Mehsana, Pandharpuri, Surti, and Toda using ddRAD sequencing protocol. Initially, the genotypes were used to carry out population diversity and structure analysis among the six breeds, followed by pair-wise comparisons of Murrah with the other five breeds through XP-EHH and *F*_ST_ methodologies to identify regions under selection in Murrah. Admixture results showed significant levels of Murrah inheritance in all the breeds except Pandharpuri. The selection signature analysis revealed six regions in Murrah, which were identified in more than one pair-wise comparison through both XP-EHH and *F*_ST_ analyses. The significant regions overlapped with QTLs for milk production, immunity, and body development traits. Genes present in these regions included *SLC37A1*, *PDE9A*, *PPBP*, *CXCL6*, *RASSF6*, *AFM*, *AFP*, *ALB*, *ANKRD17*, *CNTNAP2*, *GPC5*, *MYLK3*, and *GPT2*. These genes emerged as candidates for future polymorphism studies of adaptability and performance traits in buffaloes. The results also suggested ddRAD sequencing as a useful cost-effective alternative for whole-genome sequencing to carry out diversity analysis and discover selection signatures in Indian buffalo breeds.

## Introduction

Water buffalo is considered as an important livestock resource in tropical and sub-tropical countries due to its high milk production ability along with adaptability to hot and humid environment, and high feed conversion efficiency (Kumar et al., 2019). Buffaloes are the major contributors of milk production in India accounting for 49.2% of 187.7 million tons of total milk production (DAHD&F, 2018). India possesses a remarkably large and diverse buffalo population with 109.85 million buffaloes and 17 registered breeds (DAHD&F, 2018; NBAGR Karnal, 2021).

Murrah is the most important buffalo breed of India, constituting about 44.3% of the total buffalo population of the country. The main breeding area of this breed is the northern states of India, namely Punjab, Haryana, and Western Uttar Pradesh. Due to its high milk potential in varied environmental conditions, the germplasm of the breed has been extensively used throughout the country. It has also been imported in several countries like China, Brazil, Vietnam, Egypt, Bangladesh, etc., due to its higher milk production potential (Zhang et al., 2020). As part of the breed improvement schemes, Murrah buffalo has been selected for improved milk production for the past 30 years, and the process is going on. By investigation of selection sweeps in the Murrah genome, we may gain insights into the genes and genomic regions related to important economic traits in buffaloes. Recently, Dutta et al. (2020) identified selection sweeps in seven Indian riverine buffaloes and compared patterns of between-species selective sweeps with different cattle breeds using whole-genome sequencing (WGS) data. Since WGS is a costly process, several workers have proposed reduced representation genotyping techniques such as the double digest restriction site-associated DNA sequencing (ddRAD-seq) as a useful alternative to WGS for genotyping Indian buffaloes (Surya et al., 2019; Mishra et al., 2020). For the present study, the genotype data of six Indian buffalo breeds (Murrah, Surti, Mehsana, Bhadawari, Pandharpuri, and Toda) was generated using ddRAD sequencing.

This study aimed to assess the genetic diversity and population structure among the six Indian buffalo breeds using ddRAD data. Furthermore, we attempted to unravel signatures of positive selection in Murrah by comparing it with other reference Indian breeds (Surti, Mehsana, Bhadawari, Pandharpuri, and Toda) through cross-population extended haplotype homozygosity (XP-EHH) and cross-population fixation index (*F*_ST_) approaches.

## Material and Methods

### Sample Collection and Generation of Double Digest Restriction Site-Associated DNA Data

Ninety-six samples were collected from six breeds of riverine buffalo from different parts of India. These breeds are diverse in terms of physical features, milk production, and adaptation. Selection of the animals was done in a way to cover the genepool of the respective breeds. So the animals of all the breeds in the present study were chosen randomly from their respective institutional farms (except animals of the Toda breed of buffalo for which random samples were collected from its breeding tracts in the Nilgiri Hills area of Tamilnadu state of India). As the Murrah breed is mainly found in the northern part of India, the random samples were collected from three institutional farms of the area, i.e., the Livestock Research Station (LRS) ICAR-IVRI situated in Izatnagar, Bareilly (Uttar Pradesh), the Buffalo Farm at livestock research station of GBPUA and T, Pantnagar (Uttarakhand), and the Livestock Farm, GADVASU Ludhiana. The samples of Bhadawari buffalo were collected from the Buffalo Farm, ICAR-IGFRI, Jhansi (Uttar Pradesh), Mehsana buffalo samples were collected from the Livestock Research Station, SDAU, SK Nagar (Gujarat), Surti buffalo samples were collected from the Livestock Research Station, CVAS, Udaipur (Rajasthan), and Pandharpuri buffalo samples were collected from the Buffalo Farm, Zonal Agriculture Research Station, Kolhapur (Maharashtra). All these farms are situated in their respective breeding tract, and animals were randomly selected from these institution farms as to cover substantially the genepool of the population. The breed-wise details of sample numbers and location are also provided in Supplementary Table S1. Whole-blood samples were collected from the jugular vein of the animals in 10-ml vacutainers under aseptic condition, and genomic DNA was extracted using the standard phenol–chloroform method (Sambrook and Russell, 2006). The concentration and purity of the DNA were measured using agarose gel electrophoresis and NanoDrop spectrophotometer. Following the ddRAD protocol (Peterson et al., 2012), the double digestion of genomic DNA was carried out using Sph I and MluC I enzymes as mentioned in Kumar et al. (2020), and the samples were sequenced on Illumina Hi-seq 2000 platform to generate 150-bp reads.

### Quality Control and Variant Calling

The reads were quality checked using FastQC (Andrews, 2010). Trimming of Illumina universal adapters and quality filtering was performed by the *process_radtags* function of the STACKS v2 software (Rochette et al., 2019). Reads were examined using a sliding window spanning 15% of the read length, and the reads having average phred score of <15 were discarded. The barcode of the reads was removed using Cutadapt 2.10 (Martin, 2011).

The paired reads were aligned to the *Bubalus bubalis* assembly UOA_WB_1 downloaded from NCBI (Low et al., 2019; https://www.ncbi.nlm.nih.gov/assembly/GCF_003121395.1/) using BWA-MEM 0.7.17 (Li, 2013) with default settings. The percentage of reads aligning to the reference genome was determined by Samtools (v1.7) flagstats (Li et al., 2009) function. Variant calling was performed through the bcftools mpileup utility of the Samtoolsv1.7 suite in a multi-sample mode as recommended by Wright et al. (2019). SNPs with quality score greater than 30 and a read depth of 10 were retained for further analysis.

The structural and functional annotation of the retained SNPs was performed using SnpEff v4.3 (Cingolani et al., 2012). Quality filtering of the annotated variants was performed by removing unmapped and non-autosomal SNPs. SNPs missing in more than 25% of the individuals and below the minor allele frequency (MAF) threshold of 0.01 were also filtered out using PLINK 1.9 (Purcell et al., 2007). Genotype imputation of sporadically missing genotypes was done using Beagle 4.1 (Browning and Browning, 2016).

### Genetic Diversity and Population Structure Analysis

Linkage disequilibrium (LD) pruning of the SNPs was carried out using the *indep-pairwise* command parameters (indep-pairwise 50 5 0.2) of the PLINK software. The observed (Ho) and expected (He) heterozygosities for different buffalo breeds were estimated using PLINK 1.9. Furthermore, admixture analysis was performed on the LD pruned data for K values ranging from K = 2 to K = 6 using ADMIXTURE 1.3 software (Alexander et al., 2009). The results of the admixture analysis were visualized using PONG (Behr et al., 2016). A genomic relationship matrix was prepared in GCTA (Yang et al., 2011), and the first 10 principal components were extracted. The top principal components were plotted in R (R Core team, 2018) to visualize population clustering. A maximum-likelihood phylogram was constructed using TREEMIX (Pickrell and Pritchard, 2012) to infer the ancestral relationships and migration patterns between the breeds.

### Analysis of Selection Signatures

Cross-population selection signatures between Murrah buffalo and five other Indian water buffalo breeds (Bhadawari, Surti, Mehsana, Pandharpuri, and Toda) were derived using XP-EHH (Sabeti et al., 2007) and *F*_ST_ (Weir and Cockerham, 1984) methodologies. The genotypic data of all the breeds were phased using BEAGLE v5.1 (Browning et al., 2018) using default settings (burnin = 6; iterations = 12; and phase-states = 280). The XP-EHH scores of the Murrah buffalo were calculated for each breed comparison using the R package *rehh* (Gautier et al., 2017), taking the other water buffalo breeds in the study as the reference populations. To detect positive selection, average XP-EHH scores were computed for 100-kb regions with a 50-kb overlap. Regions with absolute XP-EHH scores of four or above were considered as putative candidate regions in Murrah.

The pairwise *F*_ST_ estimates between the Murrah and other buffalo breeds were calculated with VCFTOOLS (Danecek et al., 2011), with a sliding window of 100 kb and a 50-kb step size. Windows belonging to the top 0.1% of the *F*_ST_ values were considered as potential regions under selection (Singh et al., 2020).

The candidate genes in the selected regions were annotated using the GTF (gene transfer format) file supplied with the UOA_WB_1 assembly, using BEDTools (Quinlan and Hall, 2010) *intersect* function. Each putative selected region was cross-referenced with the literature to find previously detected regions of functional importance.

## Results

In the present study, total 397.8 million paired-end reads of 150-bp length were obtained for the 96 buffalo breeds, averaging 4.14 million reads per sample. After initial quality control, a total of 367.2 million reads (92.3% of the total reads) of average 135-bp length were retained. The average alignment rate of the reads was 99.82% with the reference genome. Sample-wise alignment percentages are given in Supplementary Table S2. A total of 569,535 variants were identified, out of which 502,476 were SNPs and 67,059 were indels. A total of 551,458 variants were present on autosomes, 15,315 on the X chromosome and 12 on the mtDNA, and 2,750 variants were located on unmapped contigs (Supplementary Table S3). A variant was discovered for every 4,637 bp of the genome length. The total number of SNPs and indels of each buffalo breed at read depth 10 is mentioned in Supplementary Table S4. The highest number of SNPs was found for the Mehsana (489,738) buffalo followed by the Murrah buffalo (484,449), and lowest for the Toda buffalo (448,714). After quality control and imputation of sporadically missing genotypes, a total of 237,762 SNPs, which were common across all the breeds, were used for downstream analysis.

### Genome-wide Annotation of SNPs in Water Buffalo Breeds

Based on the sequence ontology terms, a greater number of identified SNPs were located within the intronic regions (66.57%), followed by the intergenic regions (22.13%), and 0.34% of SNPs were found to be located in the transcript region (Supplementary Figure S1). The impact-wise and region-wise distribution of variant effects, as generated by SNPeff, are given in Supplementary Table S5.

About 71.89% of the annotated SNPs were identified as transitions (Ts) while 28.10% as transversions (Tv) with a T_S_/T_V_ ratio of 2.5578. The Ts/Tv ratio serves as a quality control indicator of high-throughput sequencing data. Our values are consistent with previous reports of targeted sequencing methods in buffalo (Surya et al., 2019; Kumar et al., 2020).

### Genetic Diversity

For the genetic diversity and population structure analyses, we used a subset of 67,798 SNPs after pruning the SNPs in LD. The average observed heterozygosity (Ho) and expected heterozygosity (He) of all breeds in the study are presented in Table 1. The Ho and He was found highest for the Murrah (0.237 and 0.246) and lowest for the Toda (0.215 and 0.211). The genetic distances (*F*_ST_) of the Murrah with the Bhadawari, Mehsana, Surti, Pandharpuri, and Toda were 0.11, 0.17, 0.09, 0.15, and 0.13, respectively.

**TABLE 1:** Number of animals, means of observed (HO) and expected heterozygosity (HE), and differentiation (FST) between each breed and the Murrah.

S.No	Breeds	Number of animals	Ho	He	*F*_ST_
1	Murrah	30	0.2372	0.2462	-
2	Bhadawari	15	0.2343	0.2366	0.11
3	Mehsana	15	0.2314	0.2239	0.17
4	Surti	15	0.2361	0.2255	0.09
5	Pandharpuri	15	0.2366	0.2390	0.15
6	Toda	6	0.2150	0.2111	0.13

### Population Structure

**FIGURE 1:**
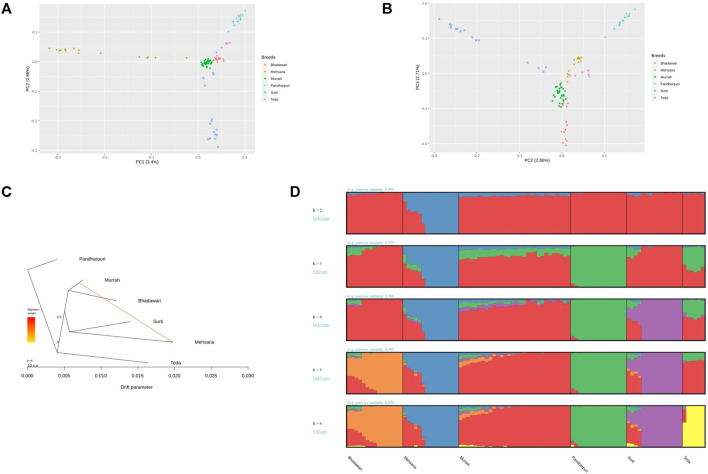
(**A**) Plot of the first two principal components (PC1 and PC2) of the genomic relationship matrix of the 96 animals under study. (**B**) Plot of PC2 and PC3. (**C**) Treemix phylogram showing one migration path. (**D**) Admixture results from K = 2 to K = 6.

The population structure of the Indian water buffalo breeds was identified using PCA. The first and second principal component (PC) explained 3.4 and 2.86% of the total variance. PC1 separated the crossbred Mehsana individuals from the rest of the breeds, while PC2 separated the Pandharpuri, Surti, and Toda from the Murrah and Bhadawari (Figure 1A). PC3 explained 2.71% of the total variation and showed clear separation between the Murrah and Bhadawari (Figure 1B).

The maximum-likelihood phylogram constructed with Treemix also displayed a similar tree (Figure 1C). The addition of one migration path in Treemix revealed the introgression of the Murrah inheritance in the Mehsana buffaloes. This tree explained 99.6% of the covariance observed between populations, whereas the tree without any migration events included explained only 98.3% of the covariance.

As seen with PC1, the Mehsana was separated from the rest of the breeds at K = 2 in the admixture analysis. K = 3 separated the Pandharpuri as a distinct population from the rest of the breeds, which gives credence to the results of the phylogenetic analyses. The Toda samples in our study showed a mixture of Pandharpuri and Murrah inheritance. At K = 6, all the breeds were assigned to their own clusters, with varying levels of Murrah ancestry appearing in other breeds (Bhadawari, Mehsana, Surti, and Toda) (Figure 1D).

### Cross-Population Signatures of Selection (XP-EHH and F_ST_)

**FIGURE 2:**
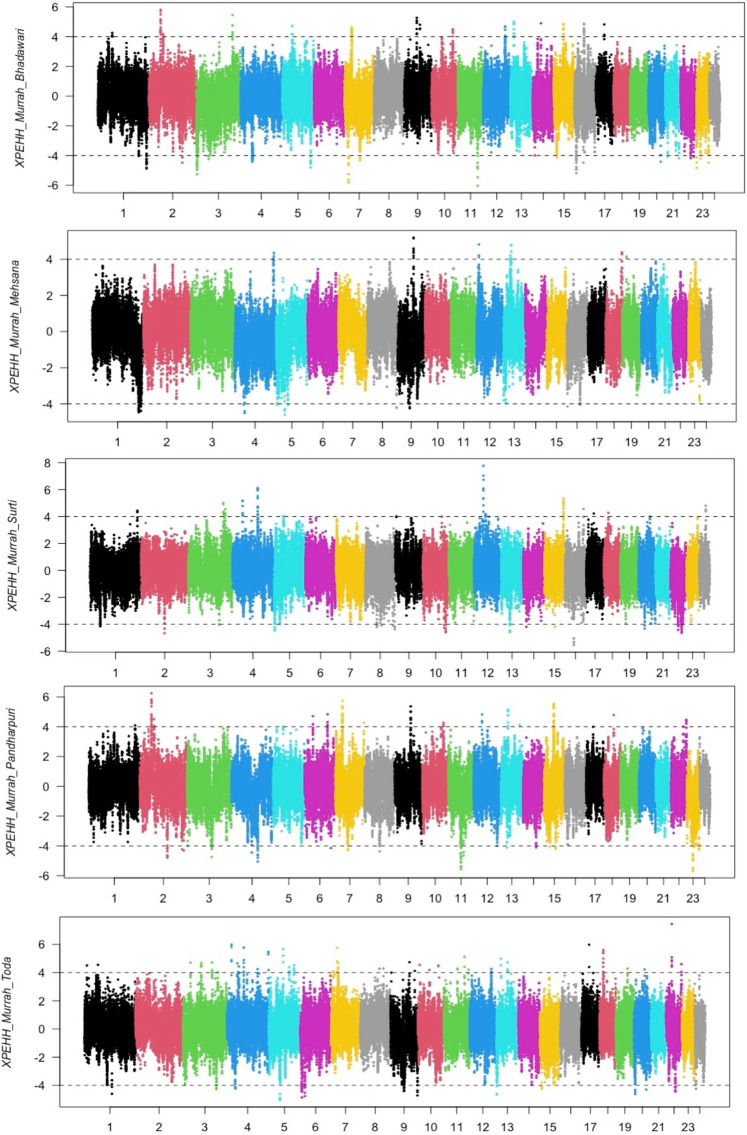
Cross-population extended haplotype heterozygote (XP-EHH) plot of the Murrah in comparison with the Bhadawari, Mehsana, Surti, Pandharpuri, and Toda.

The distribution of XP-EHH scores for the Murrah buffalo (positive values) against other water buffalo breeds in the study is visualized in Figure 2. A total of 164 putative selection regions for the Murrah buffalo were identified in comparison with the reference breeds (Supplementary Table S6). Ten selection sweeps were detected in comparisons of the Murrah with more than one breed (Table 2).

**TABLE 2:** Common selection sweeps identified by cross-population extended haplotype homozygosity (XP-EHH) in two or more pairwise comparisons involving the Murrah.

S.No	References breeds	Chr	Start	End	Annotated gene
1	Bhadawari	1	192,319,897	192,322,098	*LOC112580862*
	Mehsana				
2	Bhadawari	2	56,674,658	56,740,551	*HS6ST1*
	Pandharpuri				
3	Bhadawari	3	143,278,931	143,620,455	*DAPK1, CTSL, FBP2*
	Surti				
4	Surti	4	41,323,382	41,449,515	*IP O 8, CAPRIN2*
	Toda				
5	Bhadawari	7	28,640,078	30,146,985	*AFM, AFP, ALB*
	Toda				
	Pandharpuri				
6	Mehsana	9	64,216,990	64,326,407	*NEUROG1, TIFAB*
	Pandharpuri				
7	Bhadawari	10	84,290,283	84,562,847	*BCKDHB*
	Pandharpuri				
8	Toda	12	86,340,919	86,501,726	*KCNF1*
	Bhadawari				
9	Toda	20	49,776,417	49,968,750	*LOC112580801*
	Bhadawari				
10	Pandharpuri	23	48,880,371	49,056,564	*LOC112580801*
	Bhadawari				

**FIGURE 3:**
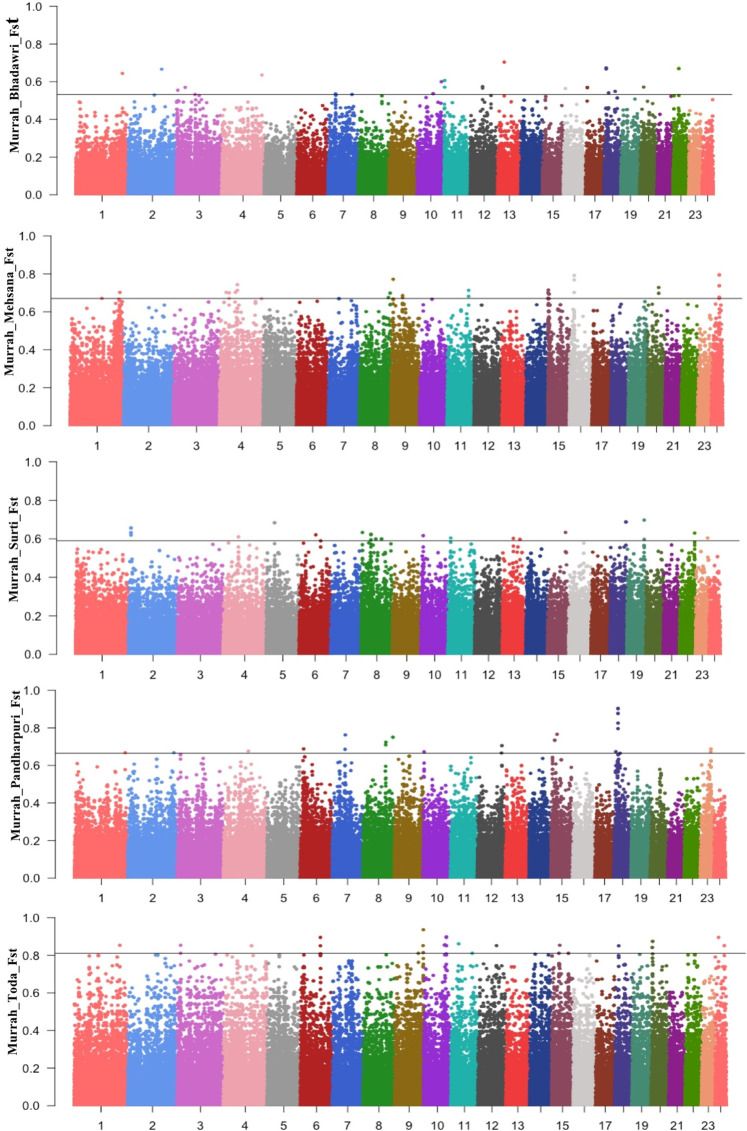
Manhattan plot for FST between the Murrah in comparison with the Bhadawari, Mehsana, Surti, Pandharpuri, and Toda.

The Manhattan plot for pairwise *F*_ST_ across all comparisons are shown in Figure 3. A total of 58 positive regions were identified from all comparisons. The selection sweeps were located on all autosomes except for chromosome 5, 14, and 21. The highest number of selected regions were identified on chromosome 8 (seven regions), followed by chromosomes 1, 9, and 10 from all pairwise comparisons (Supplementary Table S7).

A total of six fully or partially overlapping selection sweeps were identified from both the approaches XP-EHH and *F*_ST_ (Table 3). These regions were distributed on chromosomes 1, 7, 8, 13, 15, and 18.

**TABLE 3:** Selection signatures in the Murrah identified by both XP-EHH and *F*_ST_ approaches.

S. No	Test	Chr	Start	End	Genes
1	XP-EHH (Surti); *F*_ST_ (Mehsana)	1	187,322,925	187,600,000	*SLC37A1, PDE9A*
2	XP-EHH (Pandharpuri, Bhadawri, Toda); *F*_ST_ (Bhadawari)	7	28,553,887	29,108,103	*PPBP, CXCL6, RASSF6, AFM, AFP, ALB, ANKRD17*
3	XP-EHH (Surti); *F*_ST_ (Mehsana)	8	109,432,200	1,117,495,711	*CNTNAP2*
4	XP-EHH(Pandharpuri); *F*_ST_ (Bhadawari)	13	23,401,830	24,977,050	*GPC5*
5	XP-EHH (Pandharpuri); *F*_ST_ (Pandharpuri)	15	22,545,641	22,557,701	*LOC112579137*
6	XP-EHH (Toda); *F*_ST_ (Pandharpuri)	18	14,622,913	14,929,335	*C18H16orf87, MYLK3, GPT2*

## Discussion

In the present study, ddRAD sequencing was used to identify genetic variants in six water buffalo breeds of India. The average heterozygosity levels ranged from 0.215 to 0.237, which were lower compared with a previous study (Kumar et al., 2006). However, they used microsatellite data, which suffers from ascertainment bias due to the most polymorphic microsatellite markers being studied, resulting in inflated heterozygosity estimates (Fischer et al., 2017). The population structure analysis separated the six breeds under study. Our findings confirmed two existing notions about the Indian buffaloes. First, it has been traditionally believed that the Mehsana breed is of the Murrah and Surti lineage (Patel et al., 2017; Sathwara et al., 2020). The maximum-likelihood phylogram constructed using Treemix in our study showed the Mehsana and Surti emerging from the same node in the phylogenetic tree, with introgression of the Murrah germplasm into the Mehsana, which supports the anecdotal knowledge about this breed. The admixture analyses also showed varying levels of Murrah inheritance into the Mehsana breed. Second, the western Indian buffalo, the Pandharpuri, formed a separate lineage from the rest of the breeds and appeared free of any Murrah inheritance, which was in agreement with previous studies (Kumar et al., 2006). However, in our study, the geographically distinct semi-wild Toda breed clustered with the Murrah. Admixture analysis showed all the Toda samples to contain significant levels of Murrah inheritance, which is a cause for concern. The samples were collected from the hamlets of the Toda tribes, situated in the jungles in and around Nilgiris district. In the 1990s, some of the Murrah bulls were introduced in Toda hamlets near small towns. This may be one of the reasons for the inheritance of the Murrah in Toda, which is reflected predominantly due to only six samples taken in the study.

The second objective of this study was to identify positive signatures of selection in the Murrah buffaloes. Humans have exerted strong artificial selection on different breeds of buffalo for similar traits since domestication (Dutta et al., 2020). Probably milk production formed the basis of selection and breeding, which resulted in the evolution of the dairy breeds of the farmers of riverine buffalo like the Murrah, Bhadawari, Mehsana, Surti, Pandharpuri, etc. (CIRB, Hisar, 2017). The Toda, on the other end is a semi wild breed purposely used for religious values from the past in the Nilgiri hills. These breeds may share mutations in the same gene(s) or regulatory region and, consequently, may have selective sweeps in the same area of the genome. However, the scope of selective sweeps may differ among breeds sharing mutations in the same genes because of differences in breed history, effective population size, and mutation rate (Pollinger et al., 2005), and also, differences may be caused by large environmental variations and different managemental practices throughout the country.

The positive signatures of selection in the Murrah buffaloes were identified using XP-EHH and *F*_ST_ approaches. Several fully or partially overlapping candidate regions in Murrah were identified through XP-EHH comparisons against more than one breed, which indicated recent artificial selection in the Murrah, given the characteristics of the XP-EHH test (Cheruiyot et al., 2018). Many of these regions overlap with previous reports in the Murrah.

On chromosome 1, a region was identified around the 192.2 Mb position against the Bhadawari, Mehsana, and Toda, which was in agreement with Dutta et al. (2020). This region includes *UPK1B* (Uroplakin 1 B), *B4GALT4* (Beta-1,4-Galactosyltransferase), and *ARHGAP31* (Rho GTPase-activating protein 31) genes, which could be putative candidate genes undergoing selection in the Murrah. The *UPK1B* and *ARHGAP31*genes have previously been linked with growth and carcass traits in cattle breeds (Kim et al., 2012; Medeiros de Oliveira Silva et al., 2017). Another partly overlapping region (17.4–17.5 Mb) in agreement with Dutta et al. (2020) was identified on chromosome two against the Pandharpuri. The region includes *FABP3* (fatty acid-binding protein 3) gene, which is involved in the synthesis of long-chain fatty acid and, thus, regulates milk fat composition (Li et al., 2014).

A selection sweep (28.5–29.1 Mb) on chromosome seven in comparisons of the Murrah with the Pandharpuri, Toda, and Bhadawari also confirms a previously reported selection sweep (chromosome 7, 26.5–30.5 Mb) in the Murrah genome by Dutta et al. (2020). This region contains *ALB*, *AFP*, and *AFM* belonging to the family of albumin genes. The *ALB* (albumin) gene encodes albumin protein, which is involved in the transportation of varied endogenous molecules. *ALB* was reported to be significantly associated with total milk yield, milk fat, and protein percentage in the Holstein cattle (Seo et al., 2016) and obesity in humans (Kunej et al., 2013).

In agreement with Dutta et al. (2020), two regions on chromosome 13 (23.4–24.9 Mb) and chromosome 18 (14.6–14.9 Mb) were identified in our study. The region on chromosome 13 included *GPC5* (glypican 5) gene, which is linked with fatty acid composition (Li et al., 2014), fertility traits (Purfield et al., 2019), and feed efficiency (Serão et al., 2013) in cattle. The *MYLK3* (myosin light chain kinase 3) and *GPT2* (glutamic pyruvic transaminase 2) genes on chromosome 18 are involved in muscle cell development (Silva-Vignato et al., 2019; Cheng et al., 2020) and Ca^+2^ signaling pathway in contraction of striated muscles (Zhang et al., 2009).

In addition, several novel regions of positive selection were also identified. These regions contain candidate genes, which are associated with the phenotypes that are under selection in the Murrah buffalo, including milk production and fat metabolism (*HS6ST1*, *FBP2*, and *PDE9A*), immunity-related pathways (*DAPK1*), stature (*CTSL*), and fertility traits (*KCNF1* and *CNTNAP2*) (Jiang et al., 2011; Abo-Ismail et al., 2017; Guan et al., 2020). The regions included *HS6ST1* (heparin sulfate 6-O sulfotransferase 1) gene located on chromosome 2, which plays a pivotal role in heparin metabolism pathway and regulates the fatty acid composition (Jiang et al., 2011). Another region on chromosome 3 contains *DAPK1* (death-associated protein kinase 1), *CTSL* (cathepsin L), and *FBP2* (fructose bisphophatase 2) genes, which are involved in various metabolic processes such as immunity and milk production (Vineeth et al., 2019; Guan et al., 2020). The *KCNF1* (potassium voltage-gated channel modifier subfamily F member 1) gene on chromosome 12 has been previously reported to be associated with fertility traits in buffaloes (de Araujo Neto et al., 2020). Another candidate region spanning 280 kb on chromosome 1, which was detected by both approaches, contains *PDE9A* gene (phosphodiesterase 9A). This gene is involved in the signaling pathway, which regulates the level of cGMP inside the cell. Yang et al. (2015) has reported the strong association of *PDE9A* gene with milk production in Chinese Holstein cattle. On chromosome 8, *CNTNAP2* (contactin-associated protein 2) gene was present in a significant region. This gene has been reported to be associated with immunity and growth traits in cattle (Abo-Ismail et al., 2017). *CNTNAP2* gene is also reported to play an important role in milk synthesis pathway in water buffalo (Mishra et al., 2020). These positively selected genes may create the observed differences in the Murrah buffaloes from the rest of the buffalo breeds included in the study and makes the Murrah as one of the high milk-producing buffalo breed with high fertility and immunity.

## Conclusion

The genetic diversity and population structure analysis revealed varying levels of the Murrah inheritance in the Bhadawari, Mehsana, Surti, and Toda buffalo breeds. The selection signature analysis provides several genomic regions as selection signature in the Murrah, which is the prominent milch breed in India. Using reduced representation ddRAD data, our results confirm many regions, which have been previously identified as selection sweeps in the Murrah genome using WGS data. In addition, novel regions were also identified, which are involved in several biological pathways. The candidate genes, found to be positively selected, are involved in milk production (*ALB*, *FBP2*, *PDE9A*, and *GPC5*), immunity-related traits (*DAPK1*), muscle cell development (*MYLK3* and *GPT2*), and fertility traits (*KCNF1* and *CNTNAP2*). These genes are suitable candidates for future polymorphism studies to detect causative variants associated with these phenotypes in buffaloes.

## Data Availability

The genotypes of the 96 individuals under study have been uploaded to Figshare under the DOI https://doi.org/10.6084/m9.figshare.14130389.v1. The data will be made public upon acceptance of the article.
