# Supplementary figures and images for: Comparative Signatures of Selection Analyses Identify Loci Under Positive Selection in the Murrah Buffalo of India
Source: Front Genet. 2021 Oct 19;12:673697. doi: 10.3389/fgene.2021.673697 (PMC8560740; doi:10.3389/fgene.2021.673697)

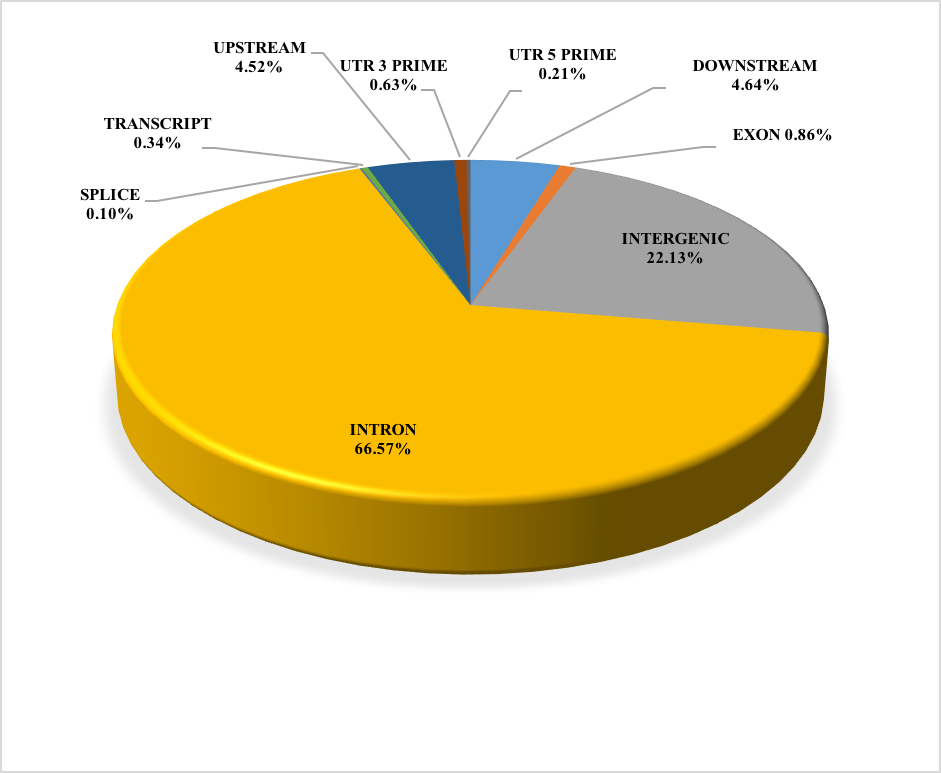

Supplement: Supplementary file 1 [file Image1.PNG]
